# Characterization of Microplastics Released Based on Polyester Fabric Construction during Washing and Drying

**DOI:** 10.3390/polym13244277

**Published:** 2021-12-07

**Authors:** Sola Choi, Miyeon Kwon, Myung-Ja Park, Juhea Kim

**Affiliations:** 1 Material & Component Convergence R&D Department, Korea Institute of Industrial Technology, Ansan 15588, Korea; solacs@kitech.re.kr (S.C.); mykwon@kitech.re.kr (M.K.); 2 Human Tech Convergence Program, Department of Clothing and Textiles, Hanyang University, Seoul 04763, Korea

**Keywords:** microplastics, microplastic fibers, polyester fabric, fabric construction, laundry

## Abstract

With the increasing production of synthetic materials, more microplastic fibers are being generated while washing clothes. Consequently, these particles are increasingly detected in the aquatic environment. Synthetic fibers produced via washing have a relatively high contribution to microplastic pollution. Hence, recent research on reducing the release of microplastic fibers is attracting considerable attention. In this study, fabric-specific analysis was performed by strictly controlling various factors, and each washing and drying process was improved by focusing on the mechanical factors affecting microplastic release. Furthermore, the mass of the collected microplastic fibers and their length distribution were measured. Fabric construction, including chemical composition and yarn type, impacted the microplastics released during washing and drying. Differences in the mechanical factors during washing helped to identify the physical factors affecting microplastic release. These results on the release of microplastics may provide a basis for developing a filter system that can minimize the unintended environmental consequences.

## 1. Introduction

Plastics detected in the aquatic environment were first investigated in 1972, when plastics were rarely used [1]. Plastic materials constitute up to 95% of the debris that pollutes marine environments, caused by the unintentional release or indiscriminate disposal of synthetic materials. This plastic debris exists in various forms, types, sizes, and colors, and has become a subject of scientific discussion [2,3,4]. Plastic waste fragmented through various processes causes marine pollution. These fragments, called microplastics, refer to a material composed of small or fine solid particles, which are made of synthetic polymers and are smaller than 5 mm [5]. Among the primary microplastics introduced into the oceans worldwide, synthetic fiber wash contributes to as much as 34.8%; hence, research is being directed toward the fiber form of microplastics [6].

The direct estimation of washing effluent as a source of microplastic fibers was first attempted in 2011 [7], and quantifying its release during washing has been researched since 2015 [8].The amount of microplastic fiber generated during fabric washing is influenced by many factors such as fabric construction (woven, knit, or non-woven), yarn type (twist, evenness, hairiness, and number of fibers), processing history (spinning, knitting, or weaving, scouring, bleaching, dyeing, finishing, and drying processes), and fiber physicochemical properties [9]. Polyester-based fabrics have been the most studied, and were selected to compare the different chemical compositions. In a study by Napper in 2016 microplastics released from three types of jumpers with different chemical compositions varied, with different fabrics releasing varying amounts of microplastic in the decreasing order of polyester, acrylic, and polyester/cotton. Unfortunately, it was difficult to determine the differences due to chemical composition because of the unavailability of accurate descriptions of the fabric parameters [10]. Recently, researchers have attempted to compare fabrics with different chemical compositions and the same weaving method, such as polyester with plain weave, polyamide with plain weave, and acetate with twill weave, but other variables could not be completely controlled [11]. Hence to ascertain the significance of a certain variable on different fabrics, other variables must be controlled. Almroth et al. (2018) studied the amounts of microplastics released from fabrics with different chemical compositions by controlling other variables such as yarn and knitting methods. However, a laboratory washing machine which exerts different mechanical forces compared to that of a large-load washing machine was used to confirm these release tendencies [12].

Factors affecting washability are classified into mechanical and physicochemical factors. When washing with detergents that are considered a chemical factor, they remove or dissolve the contaminants adsorbed on the surface, which may damage the fabric and release microplastic fibers during this process. In addition, the fabric can be damaged by mechanical action; rotation of the chamber exerts a physical force on to the fabric and increases the detergent action. Therefore, to reduce microplastics release, further experiments using different washing conditions with varied mechanical and physiochemical actions are required. In previous studies concerning microplastics released during washing, detergents and fabric softeners used were considered chemical factors [10], and the number of sequential washing cycles and the type of washing machine were considered mechanical factors [13].

Microplastic fibers released during synthetic fabric washing are the primary source of microplastic pollution. Hence, it is essential to develop techniques with different washing conditions and varied mechanical actions to help minimize the levels of microplastics released. This study focused on different synthetic fabrics that released fewer microplastics, and the microplastics released during machine washing and tumble drying were gravimetrically and microscopically analyzed. The aims of our study were: (1) to quantify the microfibers released from different types of fabric by controlling other textile parameters such as chemical composition and yarn types; (2) to observe the variation in the amount of microplastics generated during each washing procedure by individually collecting and filtering the entire wastewater effluent generated from each washing procedure; and (3) to detect microplastics that reattach to the fabric after washing, during the drying process. Focusing on fabrics by strictly controlling other textile parameters willhelp to determine the factors of fabric construction that ultimately affect fiber release. Furthermore, washing or drying parameters affecting the release of microplastics were investigated.

## 2. Materials and Methods

### 2.1. Materials

**Figure 1:**
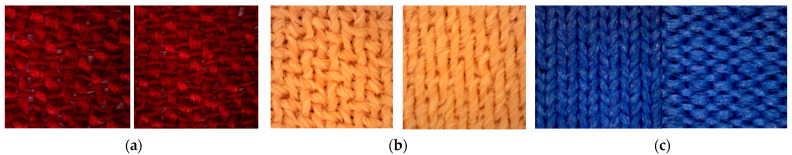
Images of three specimens with different fabric constructions taken by an image analyzer. (**a**) Plain-woven fabric; (**b**) twill-woven fabric; and (**c**) plain knit.

Samples with three different fabric constructions, but with similar chemical composition (polyester) and yarn type (filament yarn), were selected as specimens. Among the three specimens used, two were woven fabric and one was knit fabric. The two woven fabrics were plain weave and twill weave, and the knitted fabric was called plain knit, which has a basic knit construction (single jersey). The fabric density of the specimens was measured according to ISO 7211-2, and the weight (ISO 3801), thickness (ISO 4603), and size of the specimens are shown in Table 1. Confirming the polyester composition in commercially available fabrics is challenging; thus, Fourier transform infrared spectroscopy (FTIR, Vertex 80v, Bruker, Billerica, MA, USA) was used to determine the fabric composition. The fabric construction was confirmed by using an image analyzer (SU8010, Hitachi, Tokyo, Japan) (Figure 1). The plain and twill –woven fabrics were purchased from Hwan Tex (Korea), and the plain-knit fabric was purchased from B basic textile (Korea). The weight of the fabric to be tested was set to 500 g because the physical forces during washing varied depending on the fabric weight [14,15]. In this study, the experiments were performed using five pieces, each weighing 100 g. Cut edges of each prepared specimen were overlocked to prevent the loosening of the sample thread.

### 2.2. Washing and Drying

**Figure 2:**
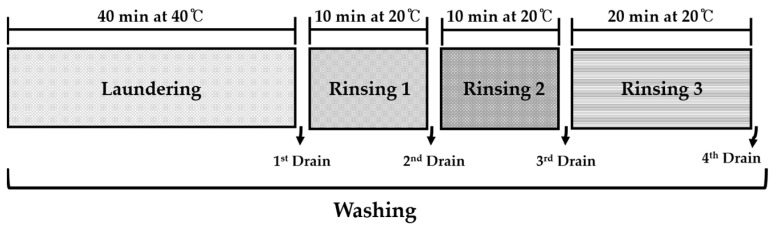
Washing procedures of a standard washing course.

Fabrics were washed in a front-loading washing machine (F9WK, LG electronics, Seoul, Korea) with a 9 kg capacity through a standard course without a dummy load. A standard washing course proceeds with one laundering and three rinsing procedures (Figure 2). During laundering, fabric specimens were washed for 40 min at 40 ± 2 °C, and for 10 to 20 min at 20 ± 2 °C during each rinsing procedure. Fabrics were washed with tap water (pH = 7) without adding detergent. Wastewater from each washing cycle was collected separately to determine the amount of microplastics generated during each procedure. After completing the experiment, the empty washing machine was washed thrice to eliminate residual microplastics or other contaminants.

Washed fabrics were dried for 100 min at 60 °C in a dual inverter heat pump-type drum dryer (RH9WGN, LG Electronics, Seoul, Korea) with a 9 kg capacity using a standard drying course immediately after washing. The drying machine was dried thrice without the fabric to ensure that no residue was retained after drying.

### 2.3. Analysis of Microplastics

The wastewater from each procedure was collected in four 20 L containers and filtered separately. Quantitative filter papers consisting of cellulose fibers with a 5 µm pore size, which is smaller than the fiber diameter, and a diameter of 185 mm (Grade 30, Hyundai micro, Seoul, Korea) were used. The wastewater filtration system was identical to that used by Choi et al. (2021) in their study [15].

Gravimetric analyses were performed and calculated using the parts per million (mg/kg) equation, which is the mass of collected microplastics per mass of the textile. Manual counting of fibers from the scaled filter paper has considerable potential for counting errors because fibrous forms, especially in microplastic fibers, are intertwined across a 3-dimensional spaghetti-like structure [16]. The filter paper’s weight was measured before and after filtering the washing wastewater, and after drying at 26 ± 2 °C and 20% RH for 24 h. The amount of microplastics released from drying was measured by comparing the built-in filter’s weight before and after drying.

The microplastic fiber length was identified using an image analyzer (magnification of 40×) and analyzed using the Image J program (NIH, National Institutes of Health, Bethesda, Rockville, MD, USA). In the washing experiments, the length of the microplastics contained in the filter paper was measured. In the drying experiment, the fibers collected from the built-in filter were shaken evenly with 100 mL of water and filtered using a 5 µm quantitative filter paper. The length of the microplastic fibers on the filter paper was analyzed the same procedure as that used for the washing experiment.

## 3. Results and Discussion

### 3.1. Release of Microplastics from Synthetic Fabrics during Washing

**Figure 3:**
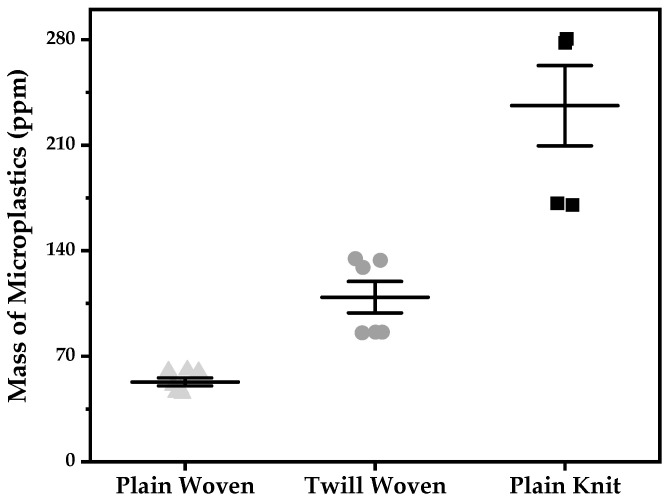
Mass of microplastics released from washing.

The results comparing the microplastics released from three different fabrics during washing are shown in Figure 3. The amount of microplastics released from plain knit fabric was higher than that of the two types of woven fabrics, and more microplastics were released from the twill-woven fabric than those from the plain-woven fabric.

**Figure 4:**
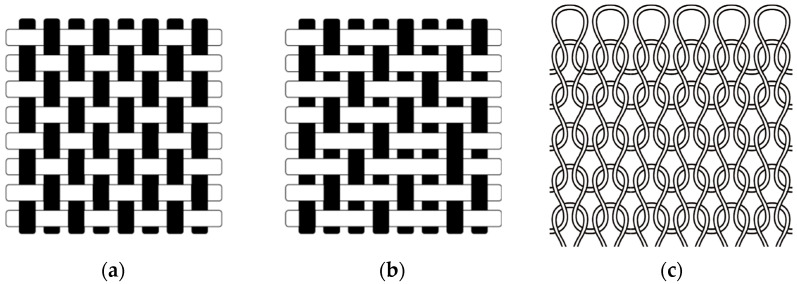
Fabric constructions of two woven fabrics and one plain-knitted fabric. (**a**) The plain-woven fabric with a 1:1 intersecting point. (**b**) Twill-woven fabric made by passing the weft thread over one or more warp threads, then under two or more warp threads. (**c**) Plain-knit fabric formed with loops that are loosely connected.

A substantial amount of microplastics was released from the knitted fabric than from the woven fabrics, which is evident because of the loose structural characteristics of the knitted fabric. The knitted fabric formed with loops is loosely connected, forming a curve, and is considerably elastic and easily deformed in the direction of the force applied on the loop (Figure 4c). Therefore, knitted fabric is susceptible to friction, has lower durability than the other fabrics, and is characterized by free movement along the intertwined fibers and yarns. Furthermore, considerable amount of microplastics released from the knitted fabric compared to the woven fabric is because its loop structure is deformed during washing. This was contrary to the result obtained by De Falco et al. (2019), who compared a double-knit jersey and plain-woven polyester fabric. Furthermore, in the study by De Falco et al. (2019), double-knit jerseys released more microplastics than those of plain-woven fabrics. This is because the yarns of the knit and woven fabric differ as single-filament yarn and ply-twisted spun yarn, respectively, and the double-knit jersey, which is a composite of two knitted fabrics, is stronger than the other knitted fabrics and is similar to the woven fabric.

Among the woven fabrics, the amount of microplastics released by the twill-woven fabric was higher than that released by the plain-woven fabric. The plain-woven fabric has more weaving points compared to the twill-woven fabric; thus, it has fewer degrees of freedom and is more rigid (Figure 4). This result corroborated with the tendencies reported by Cai et al. (2021), who used a laboratory washing machine with different mechanical forces compared to that of a large-load washing machine [17].

Therefore, from the experimental results, the higher the degree of freedom of the fiber, the higher the amount of microplastics released. These findings regarding microplastic released according to fabric construction suggest the use of fabric with higher-density threads and compact weaves during fabric manufacturing and application stages.

### 3.2. Release of Microplastics from Syntetic Fabrics during Washing Procedures

**Figure 5:**
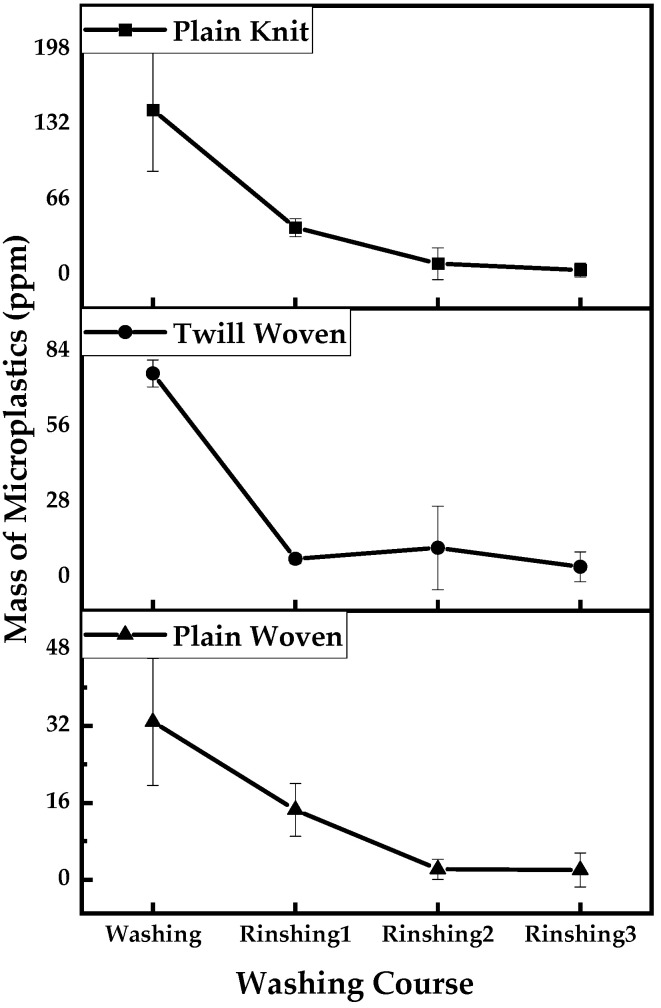
Mass of microplastics released from each washing process.

The microplastics released by each fabric exhibited identical tendencies during the washing procedures (Figure 5). Laundering produced the most microplastics, and the microplastics tended to decrease with the number of washing procedures.

Furthermore, temperature and physical forces affected the process. In other words, more microplastics were released during laundering because of the higher temperature (40 °C) and prolonged time (40 min for laundering) compared to that of the rinsing procedures (10 min for rinses 1 and 2, and 20 min for rinse 3 at 20 °C).The significance of temperature was also described in Napper and Thompson’s study (2016), where polyester released more fibers at 40 °C than at 30 °C. This provides consumers with a washing method that shortens the washing time and decreases the temperature, thereby reducing physical force.

As the washing procedure proceeded, the microplastic release decreased for all three fabrics. It can be inferred that most microplastics are removed in the initial laundering procedure and their generation gradually decreases. This is consistent with the sequential washing results of other studies. Sequential washing has been shown to consistently decrease the amount of microplastic fibers released as the number of washing cycles increases until reaching a constant level [18].

### 3.3. Release of Microplastics from Synthetic Fabrics during Drying

**Figure 6:**
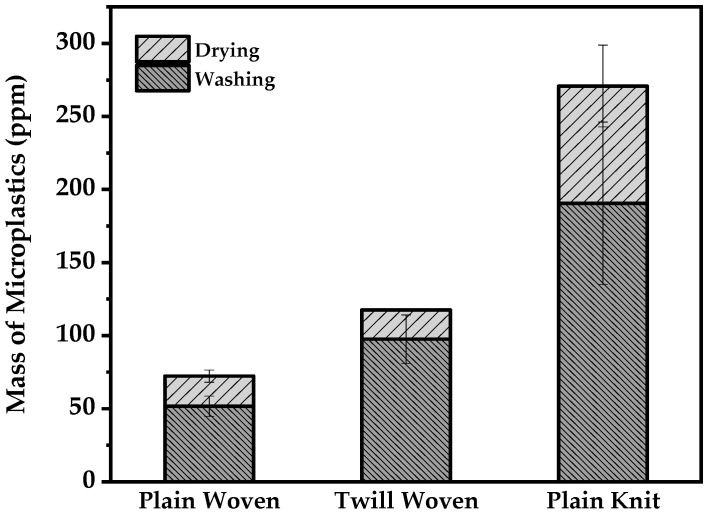
Total mass of microplastics released from washing and drying based on fabric construction.

The amount of microplastics released during drying showed similar tendencies to washing (Figure 6). The amount of microplastics released from the plain-knit fabric was higher than the two types of woven fabric. As observed during washing, drying presented the same correlation between the degrees of freedom of the fibers and microplastics released, depending on the fabric construction.

Comparing fiber emissions during washing and drying, the microplastics released was lower during drying. The release of microplastic fibers can be explained by two different mechanisms: (1) the detachment of already loose fibers from the fabric surface, which were produced during the manufacturing process of the fabric, and contained during washing; (2) washing was accompanied by additional physical forces (water) with more mechanical stress than drying. Moisture seeping into the fabric during washing makes them lose their structure, which facilitates the release of fibers. This differed from the results of Kärkkäinen and Sillanpää (2021), who demonstrated that drying was accompanied by more mechanical stress than washing.

O’Brien et al. (2020) showed that only low concentrations (1.6 ± 1.8 fibers/m^3^) of microplastic fibers were released into the room air during tumble drying and that the residence time of large particles/fibers (considerably larger than 10 µm) in the air was typically short [18,19]. Therefore, the additional drying process before and after washing suggests its use as an alternative to address microplastic emissions from wastewater effluents.

### 3.4. Length Distribution of Microplastics Released from Washing and Drying

**Figure 7:**
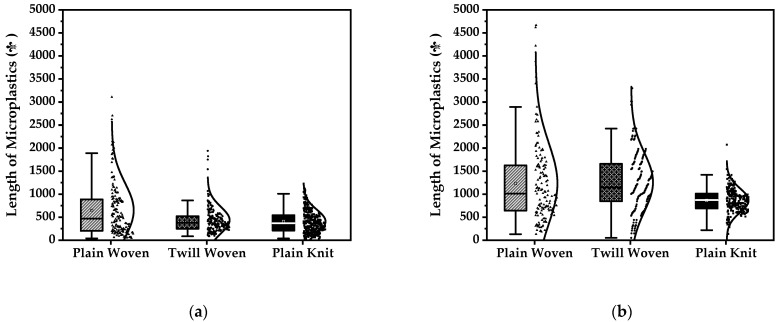
Frequency distribution diagram of the length of the microplastics. (**a**) Microplastic length distribution from washing. (**b**) Microplastic length distribution from drying.

Considering fabric construction, the fiber length distribution of microplastics released during washing and drying is shown in Figure 7. Descriptive statistics of the lengths of the released microplastic fibers are presented in Table 2. The lengths varied from the shortest fiber length of 37 µm to the longest one of approximately 4667 µm.

Based on each fabric’s construction, the microplastic lengths were similarly correlated between washing and drying. In both cases, the maximum value of fiber length was in the increasing order of plain-woven, twill-woven, and knitted fabrics. The fiber distribution in the plain-woven fabric was wider than that of the twill-woven and the plain-knit fabric, with a narrow fiber distribution in the plain-knit fabric. Therefore, the increase in the degrees of freedom of fibers based on the fabric construction increases the friction between fabrics or fibers during washing and drying, thereby facilitating the easy excision of fibers.

Comparing the fiber length of microplastics released during washing and drying, all three fabrics showed a longer length distribution during drying compared to that of washing. In the case of drying, fibers released from all three samples were more than 100 µm in length, which could be due to the difference in filter pore size between washing and drying.

## 4. Conclusions

This study proposed a method to minimize the release of microplastics. By focusing on fabric construction and strictly controlling other parameters, fabric construction was confirmed to affect the amount of microplastics released. These results of microplastics released according to the fabric construction suggest that fabrics with higher density threads and compact weaves should be used during the fabric manufacturing and application stages. The results of the washing procedures confirmed that the first laundering procedure produced the most microplastics, which tended to decrease with each washing procedure. The results of the washing procedures indicated that the release of microplastics is affected by physical factors such as temperature and time. These findings provide consumers with a more efficient washing method that shortens the washing time and decreases the temperature, thereby reducing the physical force. The amount of microplastics released during drying showed a similar tendency as that of the washing stage. The results of the tumble-drying experiments reconfirmed that fabric construction affects the amount of microplastics released, which can be proposed as an alternative for minimizing microplastics release into wastewater. The minimum length of the microplastics released during washing and drying was 37 µm and 100 µm, respectively. These results can contribute baseline data for developing optimal filter pore sizes to be used in washing machine filtration devices to efficiently collect microplastics.

## Figures and Tables

**Table 1:** Characteristics of the specimens.

Specimen	Density		Weight (g/m^2^)	Thickness (mm)	Size (m × m per 100 g)
	e.p.i/WPI ^1^	p.p.i/CPI ^2^			
Plain Woven	123.0	88.9	115.0	0.27	1.40 × 0.65
Twill Woven	137.4	81.4	207.0	0.55	1.64 × 1.88
Plain Knit (Single Jersey)	56.1	100.6	186.0	0.42	1.50 × 0.31

^1^: Ends per inch is the number of warp threads per inch of the woven fabric; WPI: wales per inch. ^2^: picks per inch is the number of warp threads per inch of the woven fabric; CPI: course per inch.

**Table 2:** Descriptive statistics of the length of the released microplastic fibers.

	Specimen	Length (µm)						
		n	*M*	*SD*	*Md*	*SE*	Min.	Max.
Washing	Plain-Woven	161	648.97	591.54	470.03	46.62	40.49	3108.03
	Twill-Woven	185	436.77	285.11	377.12	20.96	86.64	1939.31
	Plain-Knit	306	408.35	253.80	370.07	14.51	37.54	1227.44
Drying	Plain-Woven	144	1232.79	875.41	1013.48	72.95	131.75	4666.51
	Twill-Woven	102	1266.98	633.18	1163.42	62.69	148.64	3297.45
	Plain-Knit	240	863.89	262.67	872.08	16.96	140.58	2075.79

## Data Availability

Data sharing not applicable.
